# Bidirectional modulation of evoked synaptic transmission by pulsed infrared light

**DOI:** 10.1038/s41598-022-18139-2

**Published:** 2022-08-20

**Authors:** Xuedong Zhu, Jen-Wei Lin, Michelle Y. Sander

**Affiliations:** 1 Department of Biomedical Engineering, Boston University, 44 Cummington Mall, Boston, MA 02215 USA; 2 Photonics Center, Boston University, 8 Saint Mary’s Street, Boston, MA 02215 USA; 3 Neurophotonics Center, Boston University, 24 Cummington Mall, Boston, MA 02215 USA; 4 Department of Biology, Boston University, 5 Cummington Mall, Boston, MA 02215 USA; 5 Department of Electrical and Computer Engineering, Boston University, 8 Saint Mary’s Street, Boston, MA 02215 USA; 6 Division of Materials Science and Engineering, Boston University, 15 Saint Mary’s Street, Brookline, MA 02446 USA

**Keywords:** Biological techniques, Biophysics, Neuroscience, Optics and photonics

## Abstract

Infrared (IR) neuromodulation (INM) has been demonstrated as a novel modulation modality of neuronal excitability. However, the effects of pulsed IR light on synaptic transmission have not been investigated systematically. In this report, the IR light (2 μm) is used to directly modulate evoked synaptic transmission at the crayfish opener neuromuscular junction. The extracellularly recorded terminal action potentials (tAPs) and evoked excitatory postsynaptic currents (EPSCs) modulated by localized IR light illumination (500 ms, 3–13 mW) aimed at the synapses are analyzed. The impact of a single IR light pulse on the presynaptic Ca^2+^ influx is monitored with Ca^2+^ indicators. The EPSC amplitude is enhanced, and its rising phase is accelerated under relatively low IR light power levels and localized temperature rises. Increasing the IR light power reversibly suppresses and eventually blocks the EPSCs. Meanwhile, the synaptic delay, tAP amplitude, and presynaptic Ca^2+^ influx decrease monotonously with higher IR light power. It is demonstrated for the first time that IR light illumination has bidirectional effects on evoked synaptic transmission. These results highlight the efficacy and flexibility of using pulsed IR light to directly control synaptic transmission and advance our understanding of INM of neural networks.

## Introduction

Infrared (IR) neuromodulation (INM) uses IR light pulses, typically in the wavelength range of 1400–2100 nm, to reversibly modulate neuronal and muscular activities without the need of introducing any exogenous chemical or genetic mediators^1–7^. It has multiple advantages over electrical stimulation, which include high spatial and temporal precision, contact-free modulation, and being compatible with magnetic resonance imaging. Successful demonstrations of INM in diverse biological systems highlight promising clinical applications of INM, which include cochlear protheses^8,9^, brain stimulation^10–12^, cardiac pacing^13,14^, and neural identification and monitoring during surgical procedures^15^.

Though multiple mechanisms underlying INM have been proposed, it is generally agreed that the biological responses in INM are mainly caused by the localized thermal transients generated via water and tissue absorption of pulsed IR light^16–19^. These IR light-induced thermal transients can alter cell membrane structures^20–23^, passive membrane properties^17,19,21,23–26^, ion channel kinetics and activities^27–30^, and intracellular Ca^2+^ concentrations^31–34^. These changes can in turn modulate neuronal and muscular activities. For instance, in IR nerve stimulation (INS) studies, it was found that brief and intense IR light pulses can generate capacitive currents, due to reversible changes in membrane dimensions, which can depolarize the membrane potential^17,21^. In contrast, IR nerve inhibition (INI) has been demonstrated in studies where K^+^ ion channels, such as voltage-dependent K^+^ channels^29,35^ and temperature-sensitive two-pore domain TWIK-related K^+^ (TREK) channels^30^, were activated by IR light pulses. It is worth noting that the temperature rises induced by IR light pulses^30^ as well as visible light illumination commonly used in optogenetics^36^ can concurrently trigger biophysical processes that generate opposite effects in terms of the modulation outcomes. The relative importance of individual mechanisms in INM is likely to be neuron-specific. Moreover, when IR light pulses are applied to densely packed neuronal tissues, various neuron types, including non-neuronal cells^33^, of the tissues and the different subcellular compartments of individual neurons can be illuminated simultaneously and respond differently. Given that these cellular and subcellular parts may have distinct IR light sensitivities and can play different roles in a neural circuit, understanding and predicting the outcomes of INM in complex neural tissues^12,37^ needs to take these factors into consideration.

So far, most of the INM studies have focused on the modulation of neural excitability. It is expected that the synaptic transmission will be modulated if the excitability of the presynaptic neurons is modified by IR light pulses. For example, when IR light pulses were applied to the presynaptic neuronal soma or axon of cultured rat neurons, the spontaneous inhibitory postsynaptic currents were altered in their amplitude, frequency, and decay time constant^38^. Our previous study with the crayfish neuromuscular preparation demonstrated the feasibility of changing postsynaptic potentials by modulating presynaptic axonal action potentials (APs)^39^. In the vestibular system, it has also been demonstrated that IR light pulses changed afferent nerve firing via IR light modulation of presynaptic hair cells^25,37^. However, how IR light pulses can modulate synaptic processes directly has not been investigated systematically, with existing INM studies mainly focused on spontaneous postsynaptic events. In the neuromuscular junction of *Caenorhabditis elegans*, the rate of spontaneous miniature postsynaptic currents was increased following brief IR light pulses^24^. Similar phenomena were also observed in a study with rat brain slices, namely an increase in the spontaneous postsynaptic current frequency during IR light pulses^40^. It should be noted that similar changes in spontaneous synaptic activities have been reported in studies raising the temperature of the circulating bath^41–44^, though such steady-state temperature rises differ significantly from the transient and localized temperature rises induced by an IR light pulse in terms of the spatial and temporal dynamics. While these pioneering INM studies demonstrated that IR light pulses are capable of modulating spontaneous synaptic events, an in-depth understanding of the INM of synaptic function requires a more quantitative analysis of evoked synaptic transmission with controlled experimental protocols.

In this paper, we use the crayfish opener neuromuscular preparation to investigate the IR light-mediated modulation of synaptic transmission by restricting the IR light illumination to the motor synapses. The macro-patch technique is adopted here to record the excitatory postsynaptic currents (EPSCs) and presynaptic terminal APs (tAPs) elicited by electrical stimulation under varying IR light power levels. The impact of pulsed IR light on the presynaptic Ca^2+^ influx is evaluated by Ca^2+^ indicators. Analyses of these key synaptic events provide a mechanistic framework for the understanding of the IR light-mediated modulation of synaptic transmission. Results reported here offer valuable insights into the INM of neural networks where the interpretation of network outputs should incorporate the impacts of IR light pulses on both the neuronal excitability and the synaptic transmission.

## Materials and methods

### Neuromuscular preparation and electrophysiological recording

Crayfish (*Procambarus clarkii*) of both sexes with 5–7 cm head-to-tail size were purchased from Niles Biological Supplies (Sacramento, CA) and maintained in tap water at room temperature (~ 21 °C). The opener neuromuscular preparation from the first pair of walking legs were dissected in physiological saline to expose the motor axons and the muscle fibers. In this preparation, there was no spontaneous firing because the axons were separated from the central nervous system. The control physiological saline contained (in mM): 195 NaCl, 5.4 KCl, 13.5 CaCl_2_, 2.6 MgCl_2_, 10 HEPES (pH 7.4, titrated with NaOH). The saline was circulated by a peristaltic pump (Cole-Parmer, IL, USA) at a rate of around 1 ml/min. All chemicals were purchased from Sigma-Aldrich unless specified otherwise.

**Figure 1:**
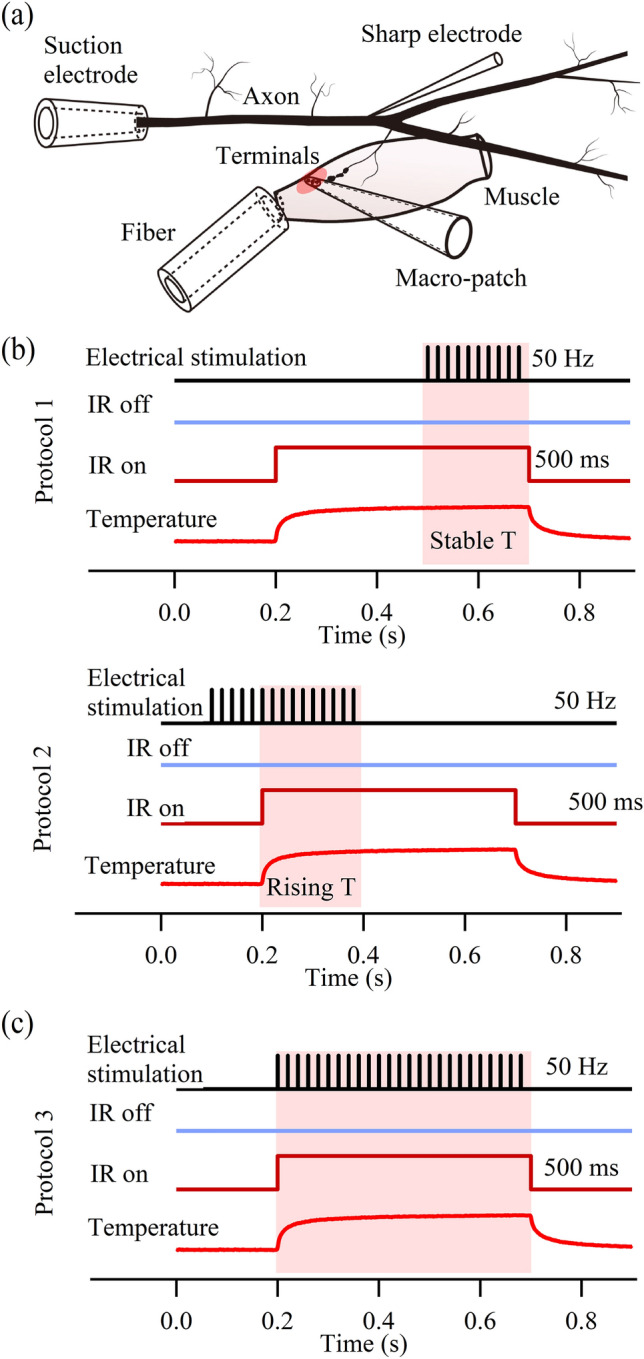
Experimental configuration and protocols. (**a**) Schematic of the electrode and fiber placements with respect to the motor axon and terminals. (**b**) Protocol 1 and Protocol 2 were used to evaluate synaptic transmission during the plateau phase (last 200 ms) and the rising phase (first 200 ms) of the IR light-induced temperature rise, respectively. (**c**) Protocol 3 was used to examine the pulsed IR light impacts on the terminal Ca^2+^ influx.

Figure 1a illustrates the experimental configuration for evaluating the INM impacts on synaptic transmission with electrophysiological recordings. A suction electrode was placed proximally to activate propagating action potentials (APs) in the excitor motor axon. Bipolar electrical pulses, 0.3 ms in duration and at a frequency of 50 Hz, from an isolated stimulator (SD9 Stimulator, Grass Instruments, RI, USA) were used to trigger a train of APs for a total of 10–15 APs. The frequency and number of the electrically stimulated APs were chosen such that sufficiently strong synaptic facilitation for the excitatory postsynaptic current (EPSC) detection was achieved while also avoiding muscle contraction. A sharp electrode penetrated the axon near the main branching point to monitor the axonal APs intracellularly. To visualize the presynaptic terminals (2–3 μm in diameter), Alexa Fluor 568 (Life Technologies Corporation, CA, USA) was injected into the inhibitory axon. Since the terminal varicosities of the excitor and inhibitor occur in pairs, the injection of the fluorescent dye into the inhibitory axon allowed for a precise localization of the excitor terminals^45^. A macro-patch pipette featuring an opening diameter of ~ 20 µm was prepared by beveling it (EG-6, Narishige) at an angle of 30° and fire polished. The smoothed rim of the pipette opening made close contact with the sample with minimal physical damage. The macro-patch pipette, filled with physiological saline, was placed over the terminal varicosities and pressed down on the muscle surface to record EPSCs^46^. A representative trace of the macro-patch recordings is shown in Supplementary Fig. S1. The electrophysiological recordings were carried out with MULTICLAMP 700B amplifier (Molecular Devices, CA, USA). Two protocols were designed for this part of the investigation. With Protocol 1 (Fig. 1b), the INM of evoked synaptic transmission was examined after the IR light-induced temperature rise had reached steady state. In this case, the AP evoked synaptic responses (EPSCs) were initiated 300 ms after the beginning of the IR light pulse. In the second protocol (Protocol 2 in Fig. 1b), the EPSCs started before the onset of the IR light pulse. This protocol allowed us to examine the INM as the temperature was rising. All recordings were performed under an upright microscope (BX51, Olympus) with a 60 × water immersion lens (LUMPLFLNN60XW, Olympus). The data acquisition and analysis were performed with Igor Pro (WaveMetrics). The voltage signals were filtered at 5 kHz and sampled at 50 kHz (NI USB-6363). Each preparation (*N*) represented a set of data recorded from the first walking leg of a crayfish. Statistical results were presented as an average ± the standard error of the mean (SEM). Samples with statistically significant differences were tested with the two-tailed Student’s *t*-test with *α* = 0.05.

### Fluorescent measurement of pre-synaptic Ca^2+^ transients

The inhibitory axon was selected for studying the Ca^2+^ transients to avoid muscle contraction that may otherwise distort the fluorescent signals, since a larger number of APs (> 25) was needed to induce a significant Ca^2+^ influx. The paired excitor axon was silenced for the same reason with intracellularly injected QX-314, which is a blocker of voltage-activated Na^+^ channels. Previous imaging and electrophysiological studies have shown that the inhibitory and excitatory axons are similar in basic structural and physiological properties^47,48^. The inhibitory axon was penetrated around the major branching point by a sharp electrode containing 500 μM of membrane-impermeant Ca^2+^ indicator Magnesium Green (K_d_ ~ 6 μM; M3733, ThermoFisher)^49^. The dye was injected by hyperpolarizing current pulses (− 2 nA and 0.25 s at 2 Hz) until (usually after 30–40 min) the terminal varicosities were clearly visible. Calcium transients induced using Protocol 3 (Fig. 1c where the AP stimulation and the IR light delivery started simultaneously) were recorded 30 min after the dye injection using an Olympus BX51 microscope with a 60 × water immersion lens. An LED illuminator, pE-300^white^ (CoolLED, UK), provided the excitation light with remote control via a TTL trigger. The LED illumination duration of a single recording trial was 2 s, with a total illumination time typical amounting to 30 min for each preparation. The LED illumination was restricted to an area with a diameter of roughly 50 μm that contained two to three terminal varicosities. The filter set (49002, Chroma Technology) included an ET470/40 × (EX), a T495lpxr (BS), and an ET525/50m (EM). The fluorescent emission was measured by a photomultiplier tube (PMT; HC124-06, Hamamatsu). The output of the PMT was filtered with a 4-pole low pass Bessel filter (LPF-100B, Warner Instruments) with a cutoff frequency of 1 kHz and digitized at 10 kHz (NI USB-6363). The relative fluorescence changes were calculated as [*F*(*t*) − *F*_rest_]/*F*_rest_ × 100%, where *F*(*t*) was the fluorescent transient and *F*_rest_ was the background fluorescence intensity. Previous studies have shown that raising the bath temperature reduced the fluorescent light intensity due to a reduced fluorescent lifetime^50^ and an increased affinity of Ca^2+^ indicators^51^. To minimize such a potential artifact, the reduction in fluorescence intensity during the IR light illumination without electrical stimulation was subtracted from that recorded when the IR light irradiation and electrical stimulation were both active (see Supplementary Fig. S2).

### IR laser light configuration

A modulated fiber-coupled diode laser (FPL2000S, Thorlabs) with a center wavelength at 1994 nm and a 3-dB linewidth of 3.6 nm was used as the IR light illumination source. The IR light pulse was aimed directly at the recorded terminals via a delivery fiber (50-µm core diameter), which was arranged at an angle of 28° to the horizontal plane. The fiber tip, cleaved at the delivery end before each experiment, was positioned slightly above the sample surface. The beam of a red laser diode was coupled into the delivery fiber first to facilitate the visual alignment of the invisible IR laser beam. An elliptical area of approximately 100 µm × 50 µm of the sample was directly illuminated by the IR light in this configuration. The duration (500 ms) and output power (3–13 mW) of the IR light pulse were modulated with an Igor Pro (WaveMetrics) software interface and a data acquisition platform (NI USB-6363) by changing the output current of the diode driver. The IR light pulse power was measured at the delivery end of the fiber pigtail in air. The resulting fluence on the surface of the sample was estimated to be about 4.88–21.13 J/cm^2^. The IR light-induced temperature transients were monitored with an open patch pipette^17,19,27^ filled with physiological saline and positioned slightly above the sample surface around the center of the IR light illuminated area (a detailed description can be found in our previous publication^19^). The patch clamp amplifier used for the temperature transient recording was set at a 1-kHz cutoff frequency and the signal was sampled at 50 kHz. With the 500-ms IR light pulse applied in this study, the IR light-induced temperature rise reached a plateau after 200 ms, as shown in Supplementary Fig. S3. The maximum peak local temperature rise close to the illuminated sample surface was ~ 19 °C for a pulse with 13-mW power, resulting in a local temperature of around 40 °C. The local temperature reached > 95% of its final level 200 ms after the IR light onset. The modulation on the EPSCs was reversible. Each preparation was exposed to 1–4 different power levels, with each power level repeated for 50–100 trials, for a total period of 2–4 h.

## Results

### Effects of varying laser power levels on evoked synaptic transmission

We first examined the dependence of the EPSC characteristics on different IR light power levels using Protocol 1 (Fig. 1b) where a train of 10 APs was delivered when the IR light-induced temperature rise could be assumed to be in steady state. The first 5 EPSCs were generally small in amplitude because of the low release probability of the crayfish excitor synapse, as shown in Supplementary Fig. S1. The amplitude gradually increased due to strong synaptic facilitation (Fig. S4). For the analysis in this section, we averaged the last 5 EPSCs (6th–10th) under each condition.

**Figure 2:**
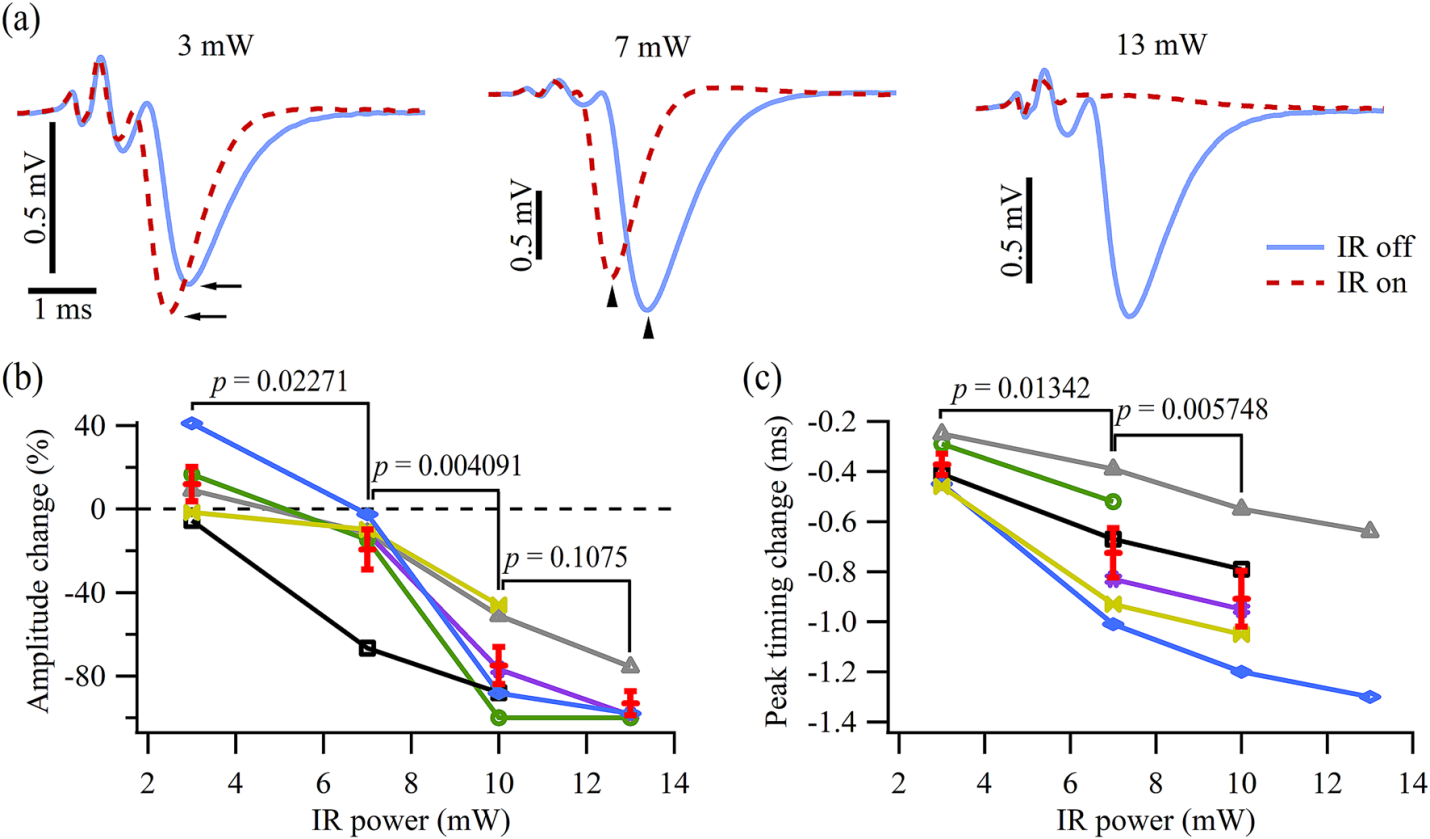
Modulation of the synaptic transmission by a single IR light pulse of different power levels. (**a**) Traces showing the EPSCs recorded from the same synapses in the absence (solid blue line) and presence of IR light illumination (dashed red line) using Protocol 1. Each trace was an average of 50 trials. All traces were recorded from the same terminal clusters. The three panels share the same horizontal scale bar on the bottom left. (**b**) Normalized EPSC amplitude changes (arrows in (**a**)) caused by the IR light of four different pulse power levels. (**c**) Differences in the timing of the EPSC peak (arrowheads in (**a**)) in response to the applied IR light pulse. Each type of symbols (colors) in (**b**) and (**c**) represents data obtained from one preparation dissected from an individual crayfish. The red bars in (**b**) and (**c**) indicate the mean ± SEM (*N* = 6).

A bidirectional modulation of the EPSC amplitude by a single IR light pulse delivered to the target synapses was achieved by varying the IR light power (Fig. 2). Specifically, the EPSC amplitude was enhanced by the IR light pulse at a low power level (Fig. 2a, 3 mW, arrows) and was suppressed by the IR light pulse of a moderate power level (7 mW). When the power level was sufficiently high, the EPSCs were completely blocked (13 mW). Additionally, the EPSCs recorded with the applied IR light pulse exhibited an earlier onset and peak than those without IR light (arrowheads in Fig. 2a, 7 mW). The localized nature of the IR light pulse illumination is supported by the observation that the intracellularly recorded APs from the main branch of the motor axons showed no change while the EPSCs recorded simultaneously were modulated by the IR light pulse (Supplementary Fig. S5) targeting the terminal varicosities. The changes in EPSC amplitude and peak timing from six preparations under different IR light power levels were summarized in Fig. 2b,c, respectively. The compiled plot showed that an IR light pulse with relatively low power enhanced or had neutral effects on the EPSC amplitude, while a pulse with higher power levels consistently suppressed and even inhibited the EPSCs (Fig. 2b). The reduction in synaptic delay (left shift of EPSC peak) continued as the IR light power increased (Fig. 2c). This bidirectional modulation of evoked synaptic transmission was completely reversible, as indicated by the stable synaptic transmission under control conditions throughout the experiment. To better examine the transition of the EPSC modulation from enhancement to inhibition, we designed Protocol 2 (Fig. 1b) where the EPSCs occurred during the rising phase of the IR light-mediated temperature rise.

**Figure 3:**
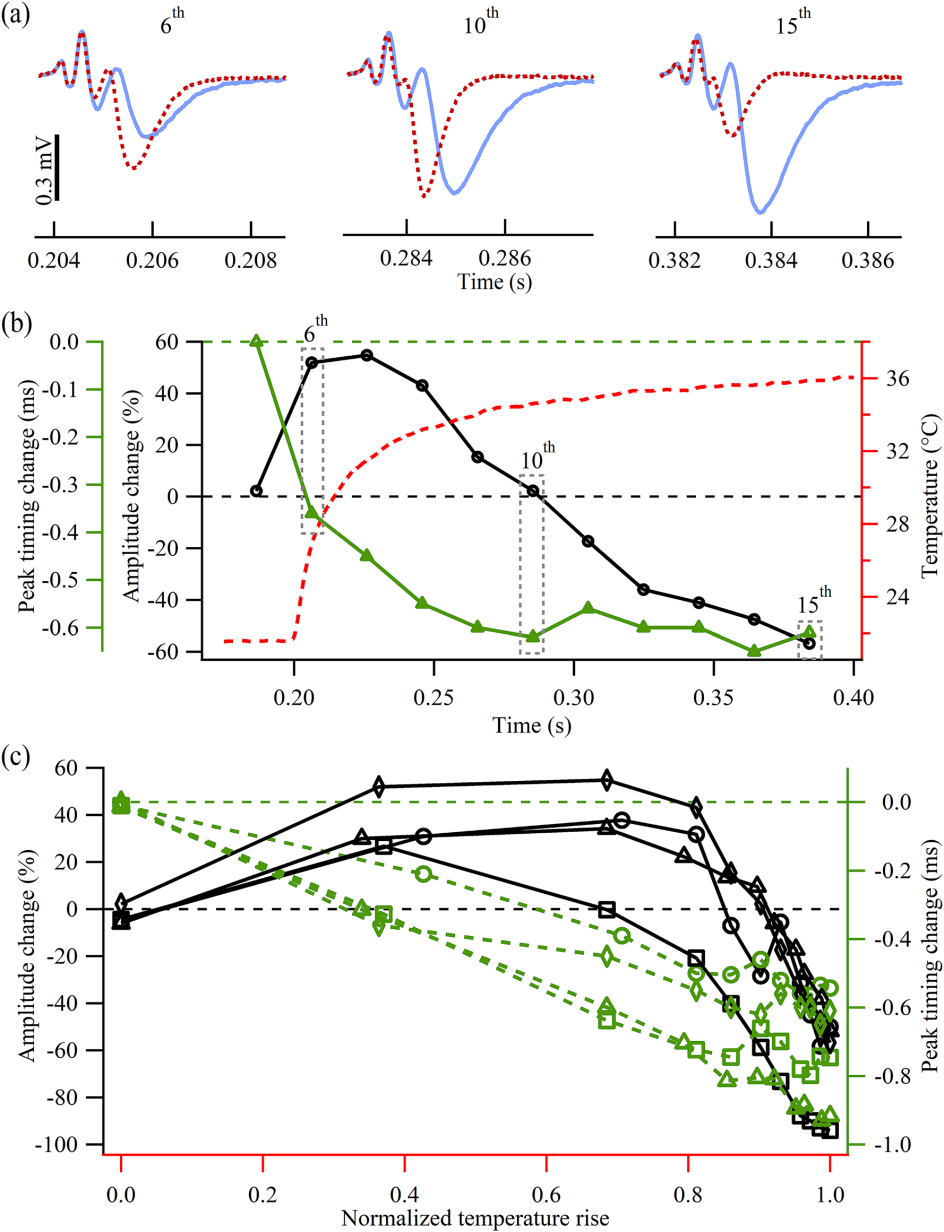
Modulation of the synaptic transmission during the rising phase of the IR light-induced temperature rise. (**a**) Traces showing the EPSCs under control (solid blue line) and IR light illumination (dashed red line) conditions with Protocol 2. The numbers (6th, 10th, and 15th) represent the order of the EPSCs within the train. Each trace was averaged over 50 trials. All traces share the same vertical scale bar on the left. (**b**) Plot showing the normalized EPSC amplitude changes (black circles), differences in the timing of the EPSC peak (green triangles), and localized temperature transient (dashed red) caused by an IR light pulse of 10 mW. (**c**) Normalized EPSC amplitude changes (left axis) and changes in the timing of the EPSC peak (right axis) in response to the normalized IR light-induced temperature rise (x axis). Each type of symbols represents a set of data obtained from one preparation dissected from an individual animal (*N* = 4).

### Synaptic transmission during the IR light-induced temperature rise

In Protocol 2, the IR light pulse started 100 ms after the onset of the electrical stimulation. The first five (1st–5th) of the 15 EPSCs before the applied IR light pulse were small because of the low release probability. The remaining EPSCs (6th–15th) were timed within the first 200 ms of the IR light pulse. Figure 3a illustrates the transition from enhancement to inhibition of the EPSC amplitude as the temperature rose after the IR light onset. Specifically, the 5th EPSC before the IR light onset remained unchanged (Fig. 3b) during the whole repeated recording session, which further confirms that the IR light effects on synapses observed here were transient and reversible. The 6th EPSC, recorded only ~ 6 ms after the IR light onset, showed a clear increase in its peak amplitude and an earlier onset. By the 10th EPSC, the EPSC amplitude was no longer enhanced while an acceleration of the peak arrival time was more pronounced than that of the 6th EPSC. The 15th EPSC amplitude was significantly suppressed and the reduction in synaptic delay remained evident. In Fig. 3b, the changes in amplitude (black y axis) and peak timing (green y axis) of the 5th (right before IR light illumination)—15th EPSCs together with the temperature rise (red y axis) induced by the IR light pulse (10 mW) were plotted. As time advanced and the temperature rose (Fig. 3b, dashed red trace), the EPSC amplitude (Fig. 3b, black circles) exhibited an initial enhancement which was followed by inhibition. EPSC peak timing gradually reduced as the temperature rose (Fig. 3b, green triangles). Consistency was verified across four preparations, as shown in Fig. 3c, where the changes in the EPSC amplitude and peak timing were plotted against the normalized temperature rise. Similar results were observed when aligning the EPSCs with the recovery phase of the IR light-induced temperature rise (Supplementary Fig. S6). Together with the data obtained from varying IR light power levels shown in Fig. 2, the results support the hypothesis that pulsed IR light can directly enhance and suppress synaptic transmission depending on the magnitude of the IR light-induced temperature rise.

### Dynamics of the EPSCs modulated by the IR light-induced temperature rise

**Figure 4:**
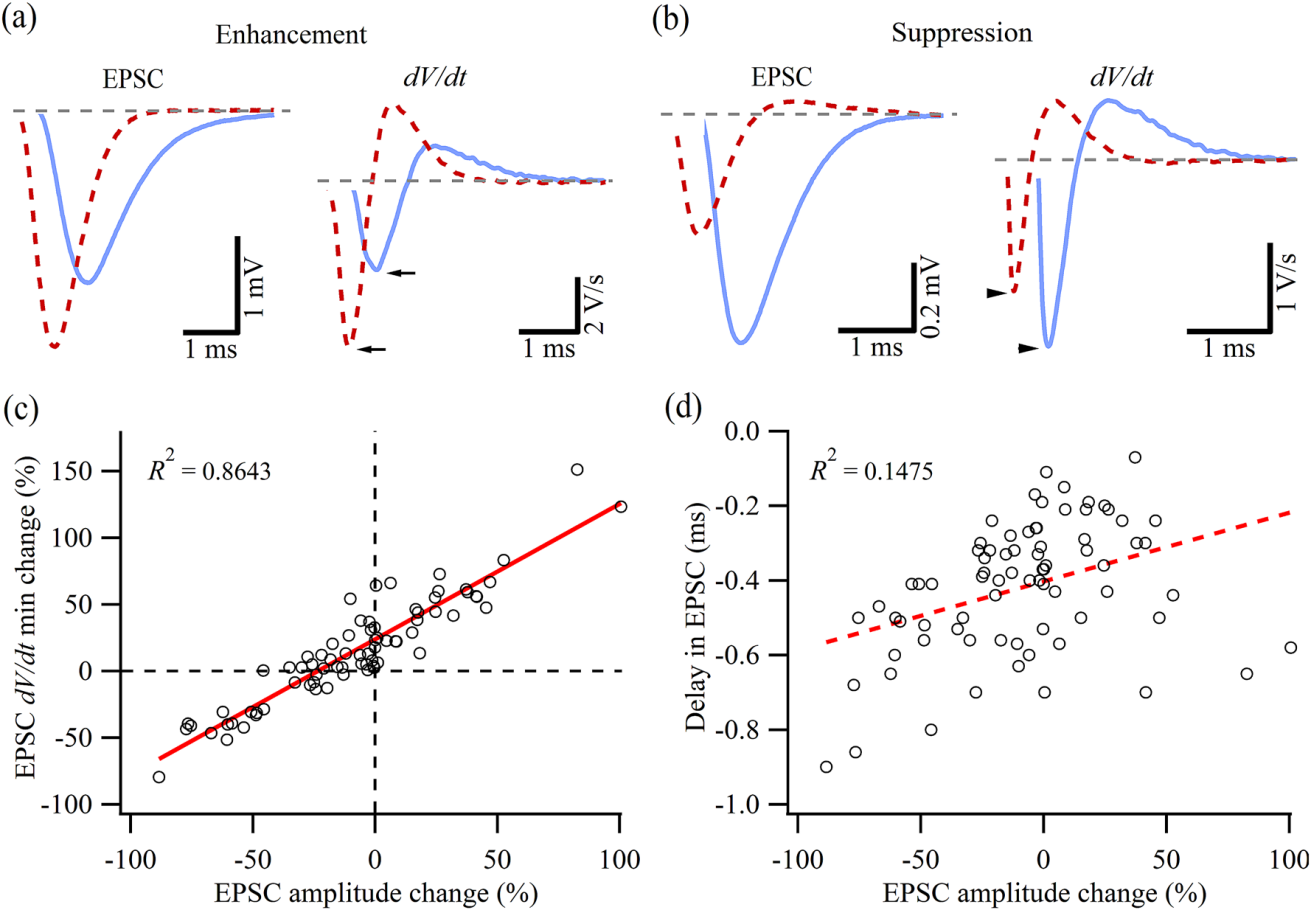
Dynamic changes of the IR light-modulated EPSCs. (**a**,**b**) Comparison of the EPSCs with (dashed red line) and without (solid blue line) IR light pulse illumination and their corresponding *dV/dt* traces for two scenarios: one with the EPSC amplitude enhanced by the IR light pulse (**a**) and the other suppressed (**b**). Each trace was averaged over 50 trials. (**c**) Scatter plot showing a positive correlation between the IR light-induced EPSC amplitude changes and the IR light-induced EPSC *dV/dt* minimum changes. (**d**) Scatter plot of the IR light-induced EPSC amplitude changes against the IR light-induced EPSC delays measured using the time when the *dV/dt* minimum occurs. Data in (**c**) and (**d**) are collected from 15 animals under different IR light power levels with Protocol 1 and Protocol 2. EPSCs whose amplitude was suppressed by more than 90% were not included in the analysis.

We further examined the IR light modulated synaptic transmission by evaluating the EPSC time course. Figure 4a,b illustrate the example EPSCs and their corresponding derivative of the voltage (*V*) with respect to time (*dV/dt*) with (dashed red) and without (solid blue) IR light illumination. The minimal peak of *dV/dt* of the enhanced EPSC was approximated two times larger than its control counterpart (see arrows in Fig. 4a). On the other hand, when the EPSC was significantly suppressed, the *dV/dt* minimum was smaller (arrowheads, Fig. 4b). We plotted the percentage changes in EPSC *dV/dt* minimum against the corresponding changes in EPSC amplitude in Fig. 4c (*N* = 15). A linear correlation between the two parameters was observed, suggesting that the two measurements accurately reflect the trend of the IR light-mediated synaptic enhancement and inhibition. All EPSCs in the presence of IR light illumination showed a reduced synaptic delay (Fig. 4d), as indicated by the measurement of the timing of the EPSC *dV/dt* minima, consistent with the data shown in Figs. 2 and 3. However, the delay in the timing of the *dV/dt* minimum and the normalized changes of the EPSC amplitude exhibited no significant correlation statistically (Fig. 4d), suggesting that the synaptic processes underlying the two parameters have different temperature sensitivities and were likely regulated individually by the IR light-induced temperature rise.

### IR light effects on terminal APs

**Figure 5:**
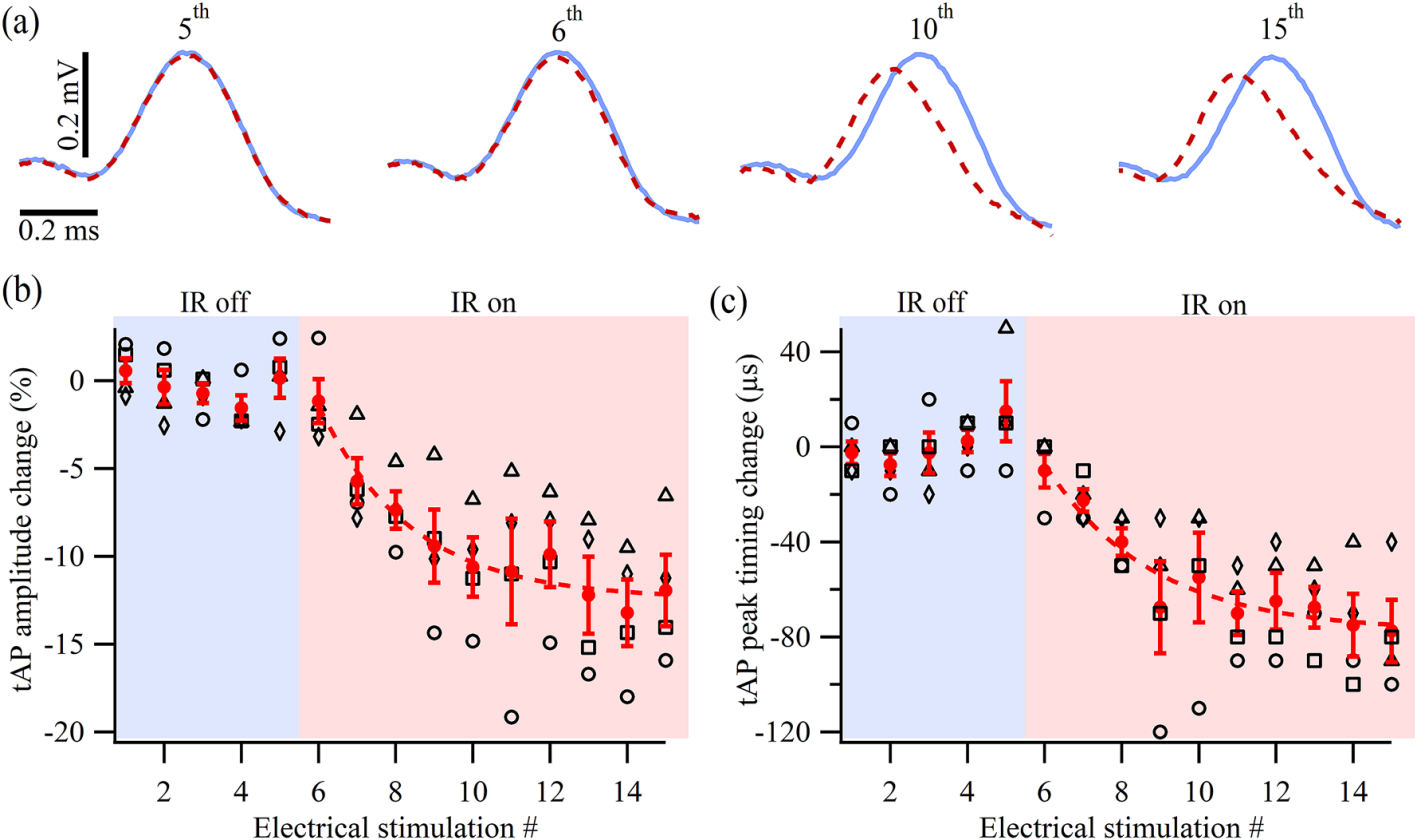
IR light effects on tAPs. (**a**) Representative examples of the tAPs, with (dashed red) and without (solid blue) IR light illumination, recorded immediately before (5th) and at various times (6th, 10th, 15th) after the onset of an IR light pulse. (**b**) Percentage changes in tAP amplitude before and during an IR light pulse (*N* = 4). (**c**) Changes in the tAP peak timing before and during an IR light pulse (*N* = 4). Each type of the black symbols in (**b**) and (**c**) represents a set of data recorded from one animal. Each solid red dot and bar represent the average ± SEM. The dashed red lines in (**b**) and (**c**) are exponential fits to the measured data.

Our previous study showed that IR light pulse can suppress the amplitude and duration of propagating APs recorded in the main trunk of the motor axon^39^. Though in the current study the axonal APs recorded around the main branching point remained completely unchanged (Supplementary Fig. S5) due to the localized nature of the IR light-induced temperature transients, the tAPs detected by the macro-patch pipette (Supplementary Fig. S1) exhibited consistent changes under different IR light power levels and local temperature rises (see Figs. 2a and 3b). The tAPs at the terminal varicosities recorded using a macro-patch pipette exhibited mainly positive transients, which is consistent with previous studies showing that Na^+^ influx in the motor axon terminals was either absent or relatively small and that APs in presynaptic terminals were mainly depolarized by Na^+^ current from proximal axonal compartments^52–55^. Figure 5a illustrates representative traces of terminal APs recorded by the macro-patch technique using Protocol 2 at different times with and without IR light illumination. Before the IR light onset, the tAPs (Fig. 5a, 5th) overlapped well. After the onset of the IR light pulse, the terminal tAPs exhibited a gradual reduction in amplitude and peak timing, as shown by the comparison of the 6th, 10th, and 15th pairs in Fig. 5a. This trend was consistent over four preparations, as shown in Fig. 5b,c, where the changes of the two parameters were plotted against the electrical stimulation (or tAP) number. The parameters measured from the first five tAPs fluctuated randomly around zero and started a downward trend when the IR light pulse was turned on between the 5th and 6th stimuli. The maximum reduction of the tAP amplitude was around 15%, which was comparable to the reduction in propagating axonal AP amplitude recorded intracellularly^39^. The maximum reduction of the tAP peak timing was ~ 80 μs. Changes in the tAP duration (full width at half maximum) were not significant.

### Effects of the IR light pulse on presynaptic Ca^2+^ influx

**Figure 6:**
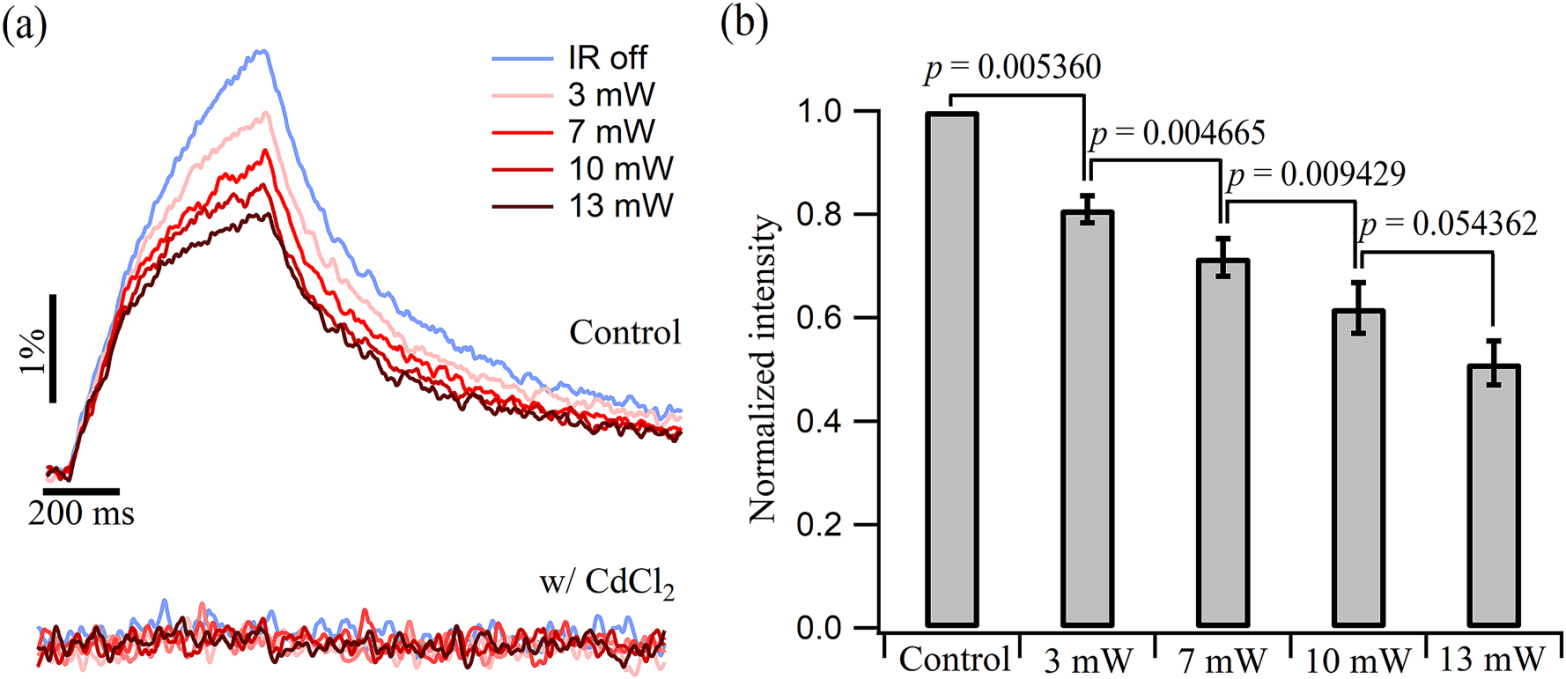
IR light-mediated presynaptic Ca^2+^ influx reduction at the axonal terminals. (**a**) Fluorescent recordings showing the AP-triggered terminal Ca^2+^ transients in response to a single IR light pulse for four different power levels with control saline (upper panel) and saline containing CdCl_2_ (bottom panel). Magnesium Green was used as an indicator of the Ca^2+^ ions. The electrical stimulation started at the same time as the IR light pulse, and both had a 500-ms duration. The data was averaged over 50 trials and filtered for presentation. The two panels share the same scales. (**b**) Bar plot showing the reduced fluorescence peak intensity caused by a single IR light pulse with four different power levels (*N* = 4).

One of the most important parameters controlling the synaptic transmission is the presynaptic Ca^2+^ influx. We evaluated the impacts of a single IR light pulse with different power levels on the presynaptic Ca^2+^ influx using Magnesium Green, a low affinity Ca^2+^ indicator. In this case, the electrical stimulation started at the onset of the IR light pulse and the two events ended both after 500 ms (Protocol 3, Fig. 1c). A train of 25 APs was fired at a frequency of 50 Hz to generate clear Ca^2+^ signals. Figure 6a shows the AP-evoked Ca^2+^ transients recorded under four IR light power levels from the same terminals in control saline (upper panel) and saline containing 200 μM CdCl_2_ (bottom panel). The IR light pulse suppressed the Ca^2+^ transient peak in a power-dependent manner. Addition of CdCl_2_ abolished any Ca^2+^ signals with and without IR light illumination. The average from four animals showed a roughly 20% reduction in the transient amplitude at 3 mW and a 50% reduction at 13 mW (Fig. 6b). Moreover, *t*-test showed that the reduction in the presynaptic Ca^2+^ influx with the 10-mW IR light pulse was not significantly different (*p* = 0.054362) from that of the 13-mW IR light pulse, which is consistent with the comparison of the EPSC amplitude changes with these two power levels presented in Fig. 2b. This indicates that the suppression in Ca^2+^ transients induced by the pulsed IR light can be an important contributor to the inhibition in the EPSC amplitude observed in Figs. 2 and 3.

## Discussion

In this report, we demonstrated for the first time that pulsed IR light can directly modulate synaptic transmission bidirectionally. Specifically, as the IR light pulse power, or the IR light-induced transient temperature rise, was slowly increased, the amplitude of the EPSCs went from being enhanced to being suppressed. For high enough IR light power, the EPSCs were reversibly and completely abolished. Meanwhile, the synaptic delay was reduced monotonously as the IR light power level was increased. We also showed that a single IR light pulse suppressed the presynaptic terminal APs and Ca^2+^ influx in a power-, or temperature-, dependent manner. These observations are further interpreted below under the hypothesis that the IR light pulse accelerated the vesicular release processes downstream to the presynaptic Ca^2+^ influx and suppressed the presynaptic terminal APs and Ca^2+^ influx. Different temperature sensitivities and dynamics of the two IR light-induced processes led to the enhancement and the inhibition of EPSCs, respectively.

### Enhancement in EPSCs

Previous INM studies have only reported IR light-induced increase in spontaneous postsynaptic events^24,38,40^. With various model systems and illumination regimes adopted, it remains unclear whether or how the synapses were directly affected by the IR light illumination. In one study, an increase in the spontaneous postsynaptic current amplitude and decay rate was observed, although the IR light pulses were delivered to the presynaptic neuronal soma or axon, not directly to the synapses^38^. Assuming the synapses were not directly affected by the IR light-induced temperature rises, the changes in spontaneous postsynaptic currents in this case could have been caused by alterations in the membrane potential of presynaptic neurons. On the other hand, if the synapses were within or very close to the IR light illuminated region^24^, an increase in the spontaneous postsynaptic current frequency could have been attributed to the temperature dependence of the presynaptic processes^41–44^.

In this study, we report that pulsed IR light at lower power levels significantly enhanced the evoked synaptic transmission. Specifically, the increase in EPSC amplitude, the reduction in synaptic delay, and the acceleration in EPSC rising phase were observed at smaller local temperature rises induced by the IR light pulse aimed at the synapses. Similar to other invertebrates, such as *Caenorhabditis elegans*^56^ and *Drosophila larvae*^57^, the excitatory synapses at the crayfish neuromuscular junctions use glutamate as neurotransmitters. Glutamate receptors in both vertebrates and invertebrates are tetramers and are structurally distinct from acetylcholine receptors at vertebrate neuromuscular junctions, which are pentamers. Thus, the discussion here will be focused on the temperature dependence of glutamatergic receptors. Studies^41,58^ using the calyx of Held to examine evoked transmitter release showed that, when the bath temperature was raised from 22–25 °C to 35–37 °C, EPSCs exhibited an increased amplitude despite a reduced presynaptic AP amplitude and duration. In the same studies, raising the bath temperature also increased the miniature EPSC amplitudes by a similar percentage as that of evoked EPSCs. It was thus suggested that the temperature dependence of the AMPA receptor activity significantly contributed to the temperature-dependent synaptic enhancement. Since the temperature range studied in this report, especially with higher IR light power levels, is similar to those using the calyx of Held, it is likely that glutamate receptors at the crayfish neuromuscular junction also responded with accelerated kinetics. However, the enhancement in EPSC amplitude reported here mainly occurred when the IR light-induced temperature rise was below 30 °C. Thus, the temperature-dependent enhancement of the postsynaptic glutamate receptor kinetics is unlikely to play a major role in the enhanced synaptic transmission when the rise in the local temperature was relatively low. Furthermore, acceleration of postsynaptic receptor kinetics cannot generate the reduction in synaptic delay that occurred simultaneously with EPSC amplitude enhancement. Nevertheless, the excitatory effects on the glutamate receptors of the crayfish neuromuscular junction were likely to be present concomitantly with, but masked by, the inhibitory processes (see below) under larger increases in local temperature.

During the enhancement in evoked EPSCs, the synaptic delay was reduced (Figs. 2 and 3), which suggests an accelerated and more synchronous vesicular release. The enhancement occurred in a temperature range where changes in the amplitude and arrival time of the tAPs were minimal (Fig. 5). Furthermore, the presynaptic Ca^2+^ transients recorded during the initial onset of the IR light pulse, the time window when the EPSC enhancement occurred, also showed no detectable change in their magnitude or their onset time (Fig. 6). Moreover, the synaptic delay continued to decrease with increasing IR light power even when an inhibition of the EPSC had become significant. In the power (or temperature) range where the EPSC inhibition occurred, the tAP amplitude and arrival time (Figs. 2a, 3a, and 5) were reduced. However, the arrival time of the tAP under IR light illumination (~ 90 µs) could not quantitatively account for the reduction in synaptic delay (up to 1 ms) at the same power levels. Thus, the presynaptic tAPs and the Ca^2+^ influx cannot account for the enhancement in the evoked synaptic transmission either.

Overall, these observations indicate that the IR light-induced acceleration of the presynaptic vesicular release processes^59^ downstream to the Ca^2+^ influx contributed significantly to the synaptic enhancement reported here. Specifically, we propose that, due to a high temperature sensitivity of the Ca^2+^-activated vesicular release processes, the IR light pulse led to an increase in the EPSC amplitude, a reduction in the synaptic delay, and an acceleration in the EPSC rising phase at relatively lower power levels and local temperature rises. These enhancements in synaptic transmission should continue to be present as the IR light power increased further, evidenced by the monotonous decrease in synaptic delay (Figs. 2 and 3). However, this enhancement was eventually dominated by the inhibitory effects as the power and the local temperature kept increasing (Figs. 2 and 3). Model systems where one could control more presynaptic parameters, such as the Calyx of Held, can further entangle these temperature-sensitive processes during the Ca^2+^-activated vesicular release.

### Inhibition in EPSCs

With increasing power levels and temperature rises, the EPSCs were profoundly suppressed and eventually blocked. To the best of our knowledge, there is no prior study that has reported inhibition of synaptic transmission by direct illumination of the synapse with IR light. Moreover, studies using mammalian synapses typically examined the synaptic transmission in the range of room to physiological temperatures and mostly observed an enhancement of the synaptic transmission near the physiological temperature^41,43,58–60^. This study reported for the first time the suppression and blocking of synaptic transmission by IR light aimed only on the synaptic terminals (Figs. 2 and 3). We observed a power-dependent suppression of tAPs (Fig. 5) and reduction in Ca^2+^ influx (Fig. 6). Since the Ca^2+^ influx is dictated by the amplitude and duration of the APs in the presynaptic terminal, it is possible that the ~ 15% reduction in tAP amplitude in combination with the altered Ca^2+^ channel kinetics can account for the ~ 50% reduction in the Ca^2+^ transients. Because Magnesium Green is a low affinity Ca^2+^ indicator, we assume the percentage reduction in the fluorescent transient amplitude is linearly correlated with the total Ca^2+^ influx activated by the AP train. Using the typical fourth-power relationship between the presynaptic terminal Ca^2+^ concentration and the neurotransmitter release, a 40–50% reduction of the Ca^2+^ transient (Fig. 6) in theory can result in an 87–94% decrease in transmitter release, which is consistent with the suppression and blocking of the evoked synaptic transmission reported here.

While the reduction in Ca^2+^ influx could quantitatively account for the inhibition of the evoked EPSCs, it remains possible that the IR light pulse can trigger other presynaptic processes that impact the synaptic transmission and result in inhibition. The range of temperature rises studied in this report fall into a regime where the membrane fluidity of the presynaptic and vesicular membranes could potentially be altered^61,62^. Proteins that constitute structural or functional components of the chemical synapses may also be modified by the induced local temperature rises, although temperature-dependent effects on specific synaptic proteins have not been reported. It is of great interest to examine these additional factors using animal models with readily attainable mutants.

### Implications

In a previous study with the same preparation, we found that, although a single localized IR light pulse can successfully suppress propagating axonal APs by reducing their amplitudes and duration, this suppression may have little effects on the downstream synaptic transmission due to the recovery of the AP waveforms once the suppressed APs propagated beyond the illuminated regions^39^. Here, we show that when the same IR light pulse was aimed directly at synapses, the synaptic transmission could be reversibly enhanced, suppressed, and blocked. The results demonstrate that synaptic transmission is highly sensitive to the IR light power and the induced local temperature rises. Therefore, the selection of the illuminated targets, even at the subcellular levels, can play a significant role in determining the outcomes of INM.

It remains to be explored how the IR light-mediated bidirectional modulation of synaptic transmission will affect mammalian synapses. It should be emphasized that the enhancement of the synaptic transmission reported here occurred mainly for an IR light pulse that raised the local temperature to below 30 °C, which is lower than the mammalian physiological temperature. The suppression and the blocking of synaptic transmission occurred at local temperatures that are close to and slightly higher than the mammalian physiological temperature. Since an IR light pulse can increase the local temperature transiently beyond the physiological level without causing tissue damage^63,64^, it would be interesting to examine the behavior of mammalian synapses when the local temperature is transiently raised from the mammalian physiological temperature, similar to what will happen during in vivo INM applications^11,12,32,34^. In addition to INM applications, the presented study also highlights the potential of pulsed IR light as a novel and convenient tool for fundamental research where transient and localized control of synaptic activity is needed.

## Conclusion

We report for the first time that pulsed IR light at lower power levels enhances the synaptic transmission while the inhibition of synaptic transmission will dominate for higher IR light power levels. The enhancement in synaptic transmission is likely due to the high temperature sensitivity of the vesicular release processes downstream to the presynaptic Ca^2+^ influx. The suppression and blocking of the synaptic transmission are mainly due to the suppression of the presynaptic terminal APs and Ca^2+^ influx. A consistent and power-dependent reduction in synaptic delay is observed across the power range tested, which is most likely caused by the enhanced efficacy of the Ca^2+^-activated vesicular release steps proposed here. Our results demonstrate the capability of the IR light pulse to bidirectionally modulate synaptic transmission, which marks a high sensitivity of the synaptic transmission to the local temperature transients generated by IR light irradiation. The presented study also highlights the need to take into account both the IR light-mediated modulation of neuronal excitability and synaptic transmission in order to fully understand the INM of neuronal tissues.

## Supplementary Information

Supplementary Figures.

## Data Availability

The datasets generated and analyzed in this study are available from the corresponding author upon reasonable request.

## References

[1] Richter C-P, Matic AI, Wells JD, Jansen ED, Walsh JT (2011). Neural stimulation with optical radiation. Laser Photonics Rev..

[2] Richter C-P, Tan X (2014). Photons and neurons. Hear. Res..

[3] Chernov M, Roe AW (2014). Infrared neural stimulation: A new stimulation tool for central nervous system applications. Neurophotonics.

[4] Zhao, K., Tan, X., Young, H. & Richter, C.-P. Stimulation of Neurons with Infrared Radiation. in *Biomedical Optics in Otorhinolaryngology* 253–284 (Springer, 2016). 10.1007/978-1-4939-1758-7_17.

[5] Hart WL, Kameneva T, Wise AK, Stoddart PR (2019). Biological considerations of optical interfaces for neuromodulation. Adv. Opt. Mater..

[6] Fekete Z, Horváth ÁC, Zátonyi A (2020). Infrared neuromodulation: A neuroengineering perspective. J. Neural Eng..

[7] Ford SM, Watanabe M, Jenkins MW (2017). A review of optical pacing with infrared light. J. Neural Eng..

[8] Matic AI (2013). Behavioral and electrophysiological responses evoked by chronic infrared neural stimulation of the cochlea. PLoS One.

[9] Thompson AC (2015). Infrared neural stimulation fails to evoke neural activity in the deaf guinea pig cochlea. Hear. Res..

[10] Cayce JM (2014). Infrared neural stimulation of primary visual cortex in non-human primates. Neuroimage.

[11] Xu AG (2019). Focal infrared neural stimulation with high-field functional MRI: A rapid way to map mesoscale brain connectomes. Sci. Adv..

[12] Horváth ÁC (2020). Infrared neural stimulation and inhibition using an implantable silicon photonic microdevice. Microsyst. Nanoeng..

[13] Jenkins MW (2010). Optical pacing of the embryonic heart. Nat. Photonics.

[14] McPheeters MT, Wang YT, Werdich AA, Jenkins MW, Laurita KR (2017). An infrared optical pacing system for screening cardiac electrophysiology in human cardiomyocytes. PLoS One.

[15] Cayce JM (2015). Infrared neural stimulation of human spinal nerve roots in vivo. Neurophotonics.

[16] Wells J (2007). Biophysical mechanisms of transient optical stimulation of peripheral nerve. Biophys. J..

[17] Shapiro MG, Homma K, Villarreal S, Richter C-P, Bezanilla F (2012). Infrared light excites cells by changing their electrical capacitance. Nat. Commun..

[18] Duke AR (2013). Transient and selective suppression of neural activity with infrared light. Sci. Rep..

[19] Zhu X, Lin J-W, Sander MY (2019). Infrared inhibition and waveform modulation of action potentials in the crayfish motor axon. Biomed. Opt. Express.

[20] Beier HT, Tolstykh GP, Musick JD, Thomas RJ, Ibey BL (2014). Plasma membrane nanoporation as a possible mechanism behind infrared excitation of cells. J. Neural Eng..

[21] Plaksin M, Shapira E, Kimmel E, Shoham S (2018). Thermal transients excite neurons through universal intramembrane mechanoelectrical effects. Phys. Rev. X.

[22] Adams WR (2022). Visualizing the lipid dynamics role in infrared neural stimulation using stimulated Raman scattering. Biophys. J..

[23] Okunade O, Santos-Sacchi J (2013). IR laser-induced perturbations of the voltage-dependent solute carrier protein SLC26a5. Biophys. J..

[24] Liu Q, Frerck MJ, Holman HA, Jorgensen EM, Rabbitt RD (2014). Exciting cell membranes with a blustering heat shock. Biophys. J..

[25] Rabbitt RD (2016). Heat pulse excitability of vestibular hair cells and afferent neurons. J. Neurophysiol..

[26] Walsh AJ, Cantu JC, Ibey BL, Beier HT, Jansen ED, Beier HT (2017). Short infrared laser pulses increase cell membrane fluidity. Optical Interactions with Tissue and Cells XXVIII.

[27] Yao J, Liu B, Qin F (2009). Rapid temperature jump by infrared diode laser irradiation for patch-clamp studies. Biophys. J..

[28] Albert ES (2012). TRPV4 channels mediate the infrared laser-evoked response in sensory neurons. J. Neurophysiol..

[29] Ganguly M (2019). Voltage-gated potassium channels are critical for infrared inhibition of action potentials: An experimental study. Neurophotonics.

[30] Zhu X, Lin J-W, Turnali A, Sander MY (2022). Single infrared light pulses induce excitatory and inhibitory neuromodulation. Biomed. Opt. Express.

[31] Dittami GM, Rajguru SM, Lasher RA, Hitchcock RW, Rabbitt RD (2011). Intracellular calcium transients evoked by pulsed infrared radiation in neonatal cardiomyocytes. J. Physiol..

[32] Cayce JM (2014). Calcium imaging of infrared-stimulated activity in rodent brain. Cell Calcium.

[33] Borrachero-Conejo AI (2020). Stimulation of water and calcium dynamics in astrocytes with pulsed infrared light. FASEB J..

[34] Kaszas A (2021). Two-photon GCaMP6f imaging of infrared neural stimulation evoked calcium signals in mouse cortical neurons in vivo. Sci. Rep..

[35] Ganguly M, Jenkins MW, Jansen ED, Chiel HJ (2019). Thermal block of action potentials is primarily due to voltage-dependent potassium currents: A modeling study. J. Neural Eng..

[36] Ait Ouares K, Beurrier C, Canepari M, Laverne G, Kuczewski N (2019). Opto nongenetics inhibition of neuronal firing. Eur. J. Neurosci..

[37] Rajguru SM (2011). Infrared photostimulation of the crista ampullaris. J. Physiol..

[38] Feng H-J (2010). Alteration of GABAergic neurotransmission by pulsed infrared laser stimulation. J. Neurosci. Methods.

[39] Zhu X, Lin J-W, Sander MY (2020). Infrared inhibition impacts on locally initiated and propagating action potentials and the downstream synaptic transmission. Neurophotonics.

[40] Entwisle B (2016). In vitro neuronal depolarization and increased synaptic activity induced by infrared neural stimulation. Biomed. Opt. Express.

[41] Kushmerick C, Renden R, von Gersdorff H (2006). Physiological temperatures reduce the rate of vesicle pool depletion and short-term depression via an acceleration of vesicle recruitment. J. Neurosci..

[42] Kim J, Connors B (2012). High temperatures alter physiological properties of pyramidal cells and inhibitory interneurons in hippocampus. Front. Cell. Neurosci..

[43] Hook MJV (2020). Temperature effects on synaptic transmission and neuronal function in the visual thalamus. PLoS One.

[44] Chen M, von Gersdorff H (2019). How to build a fast and highly sensitive sound detector that remains robust to temperature shifts. J. Neurosci..

[45] Florey E, Cahill MA (1982). The innervation pattern of crustacean skeletal muscle. Cell Tissue Res..

[46] Zucker RS (1974). Crayfish neuromuscular facilitation activated by constant presynaptic action potentials and depolarizing pulses. J. Physiol..

[47] Wright SN, Brodwick MS, Bittner GD (1996). Presynaptic calcium currents at voltage-clamped excitor and inhibitor nerve terminals of crayfish. J. Physiol..

[48] Vyshedskiy A, Lin J-W (1997). Study of the inhibitor of the crayfish neuromuscular junction by presynaptic voltage control. J. Neurophysiol..

[49] Vyshedskiy A, Lin J-W (2000). Presynaptic Ca^2+^ influx at the inhibitor of the crayfish neuromuscular junction: A photometric study at a high time resolution. J. Neurophysiol..

[50] Oliver AE, Baker GA, Fugate RD, Tablin F, Crowe JH (2000). Effects of temperature on calcium-sensitive fluorescent probes. Biophys. J..

[51] Shuttleworth TJ, Thompson JL (1991). Effect of temperature on receptor-activated changes in [Ca^2+^]_i_ and their determination using fluorescent probes. J. Biol. Chem..

[52] Lin J-W (2016). Na^+^ current in presynaptic terminals of the crayfish opener cannot initiate action potentials. J. Neurophysiol..

[53] Brigant JL, Mallart A (1982). Presynaptic currents in mouse motor endings. J. Physiol..

[54] Konishi T (1985). Electrical excitability of motor nerve terminals in the mouse. J. Physiol..

[55] Lindgren CA, Moore JW (1989). Identification of ionic currents at presynaptic nerve endings of the lizard. J. Physiol..

[56] Brockie PJ, Maricq AV (2003). Ionotropic glutamate receptors in *Caenorhabditis*
*elegans*. Neurosignals.

[57] Lnenicka GA (2020). Crayfish and Drosophila NMJs. Neurosci. Lett..

[58] Postlethwaite M, Hennig MH, Steinert JR, Graham BP, Forsythe ID (2007). Acceleration of AMPA receptor kinetics underlies temperature-dependent changes in synaptic strength at the rat calyx of Held. J. Physiol..

[59] Chao OY, Yang Y-M (2019). Timing constraints of action potential evoked Ca^2+^ current and transmitter release at a central nerve terminal. Sci. Rep..

[60] Leão RM (2005). Presynaptic Na^+^ channels: Locus, development, and recovery from inactivation at a high-fidelity synapse. J. Neurosci..

[61] Romey G, Chicheportiche R, Lazdunski M (1980). Transition temperatures of the electrical activity of ion channels in the nerve membrane. Biochim. Biophys. Acta (BBA) Biomembr..

[62] Kappel T, Anken RH, Hanke W, Rahmann H (2000). Gangliosides affect membrane-channel activities dependent on ambient temperature. Cell. Mol. Neurobiol..

[63] Liljemalm R, Nyberg T (2014). Quantification of a thermal damage threshold for astrocytes using infrared laser generated heat gradients. Ann. Biomed. Eng..

[64] Brown WGA (2020). Thermal damage threshold of neurons during infrared stimulation. Biomed. Opt. Express.

