# Supplementary material for: Bidirectional modulation of evoked synaptic transmission by pulsed infrared light
Source: Sci Rep. 2022 Aug 20;12:14196. doi: 10.1038/s41598-022-18139-2 (PMC9392733; doi:10.1038/s41598-022-18139-2)
Supplement: Supplementary file 1 — Supplementary Figures. [file 41598_2022_18139_MOESM1_ESM.pdf]

Bidirectional modulation of evoked synaptic transmission by pulsed infrared light

Xuedong Zhu[1,2,3], Jen-Wei Lin[4], and Michelle Y. Sander[1,2,3,5,6,*]

[1]Department of Biomedical Engineering, Boston University, 44 Cummington Mall, Boston, MA, 02215, USA
[2]Photonics Center, Boston University, 8 Saint Mary's Street, Boston, MA, 02215, USA
[3]Neurophotonics Center, Boston University, 24 Cummington Mall, Boston, MA, 02215, USA
[4]Department of Biology, Boston University, 5 Cummington Mall, Boston, MA, 02215, USA
[5]Department of Electrical and Computer Engineering, Boston University, 8 Saint Mary's Street, Boston, MA, 02215, USA
[6]Division of Materials Science and Engineering, Boston University, 15 Saint Mary's Street, Brookline, MA, 02446, USA
*Corresponding Author: msander@bu.edu

Supplementary materials

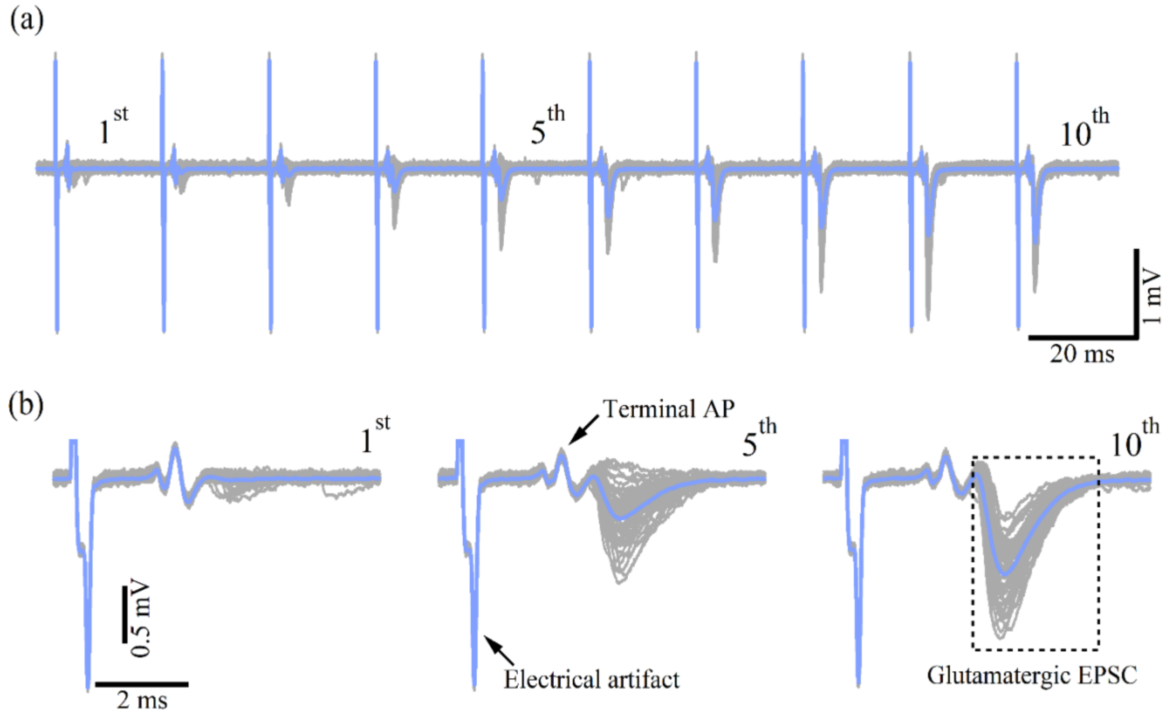

Supplementary Figure S1. Illustration of synaptic facilitation recorded with a macro-patch pipette at the crayfish neuromuscular junction. (a) The blue trace represents the averaged excitatory postsynaptic currents (EPSCs) recorded with a macro-patch pipette placed on top of the presynaptic terminals. The grey traces are recordings from individual trials. Due to the stochastic vesicle release process, it is necessary to average over 50 trials (gray traces) to obtained representative responses. (b) Zoomed-in version of three segments of the traces in (a) showing synaptic facilitation. The EPSCs (glutaminergic) gradually become larger as the stimulus number advances. The averaged blue trace is superimposed on the fluctuating individual traces (grey).

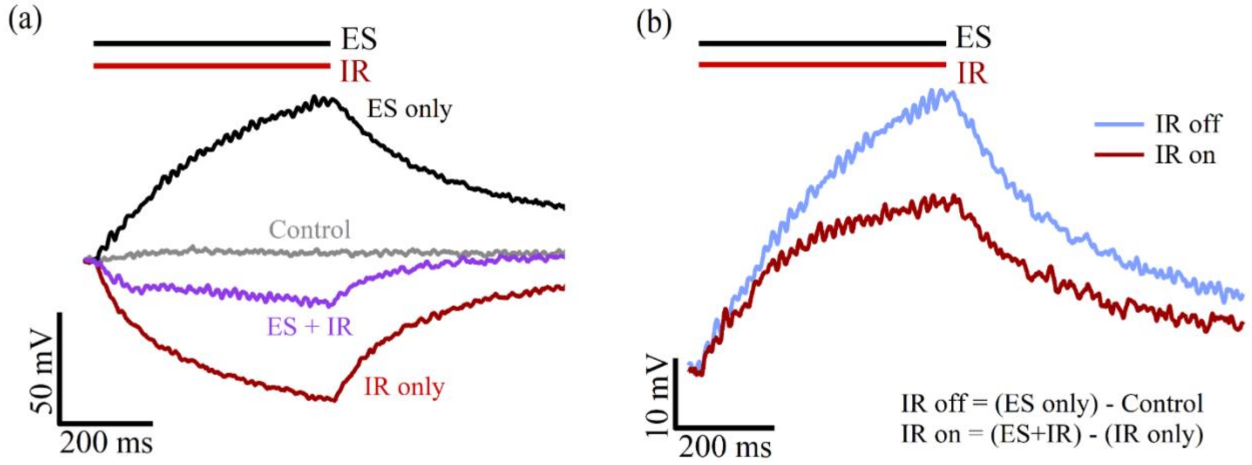

Supplementary Figure S2. Correction for the infrared (IR) light induced baseline fluorescence changes. (a) Representative raw traces of fluorescent recordings under different conditions. The reduction in baseline fluorescence intensity caused by an IR light pulse (IR only trace) was subtracted from the condition when both an IR light pulse

and electrical stimulation (ES) were applied simultaneously (ES + IR trace). (b) The corrected fluorescent transients with and without an applied IR light pulse (13 mW). Each trace is an average of 50 single recordings.

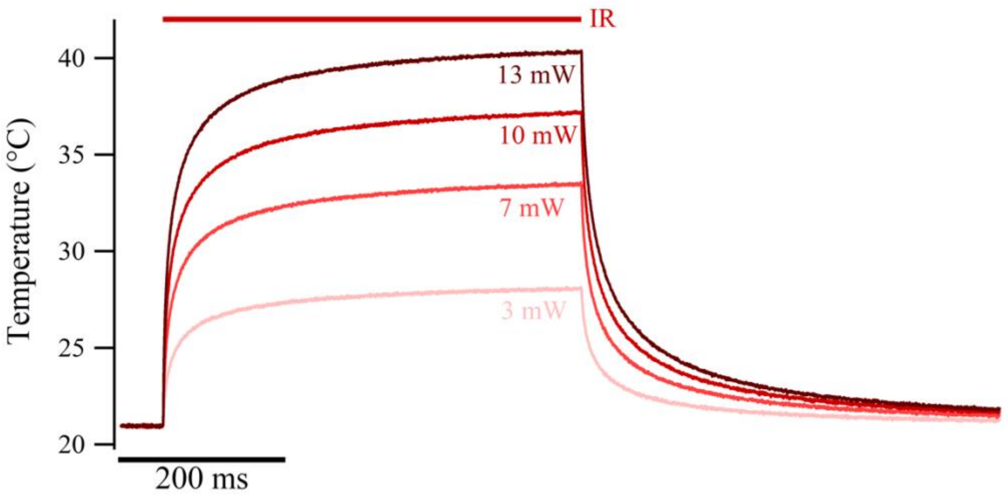

Supplementary Figure S3. IR light induced localized temperature dynamics monitored with an open patch pipette placed around the center of the illuminated area and at a distance from the optical fiber tip similar to that between the target synapes and the tip of the optical fiber.

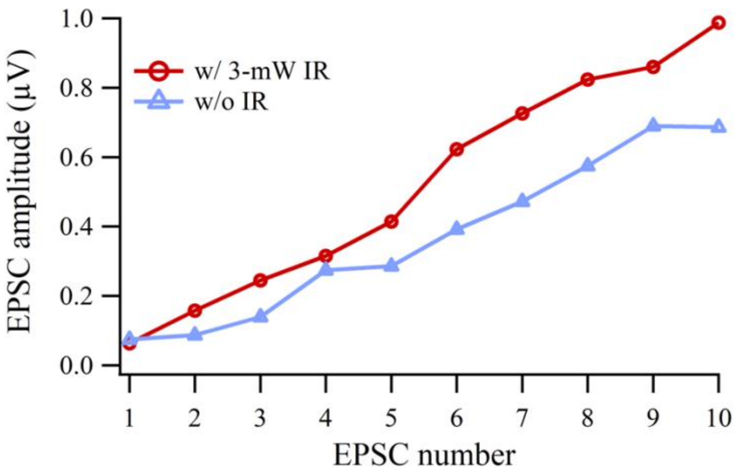

Supplementary Figure S4. The EPSC amplitude plotted against its corresponding number in the stimulation train in Protocol 1 with (red circles) and without (blue triangles) a 3-mW IR light pulse. To simplify the presentation of the results, the last 5 EPSCs in each condition were averaged for further analysis.

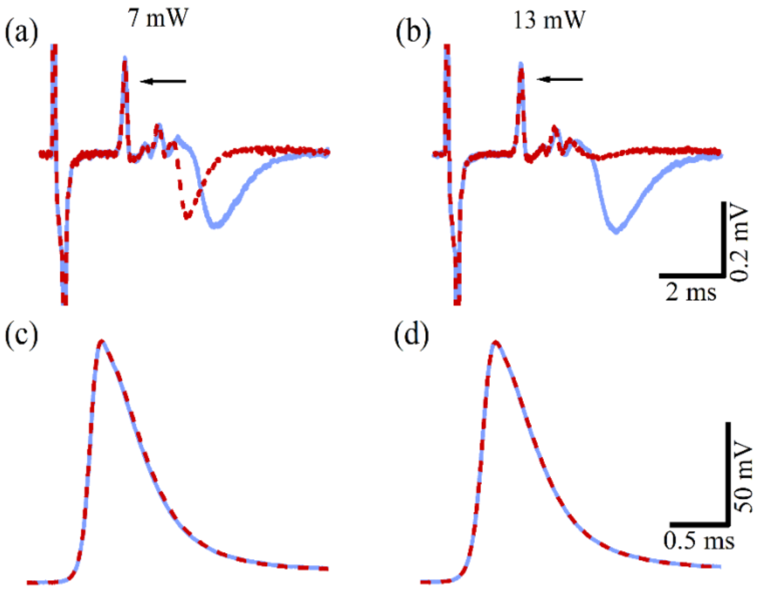

Supplementary Figure S5. Axonal APs recorded from the main trunk of the motor axons remained unchanged with an IR light pulse delivered to the terminals. (a), (b) EPSCs in response to a 7-mW and 13-mW IR light pulse, respectively (red dashed line, with IR light illumination; blue solid line, control). (c), (d) The axonal APs recorded simultaneously while a 7-mW and a 13-mW IR light pulse, respectively, were delivered to the target terminals. Arrows in (a) and (b) identify the artifacts resulting from the electric coupling of the APs from the intracellular electrode to the macro-patch pipette.

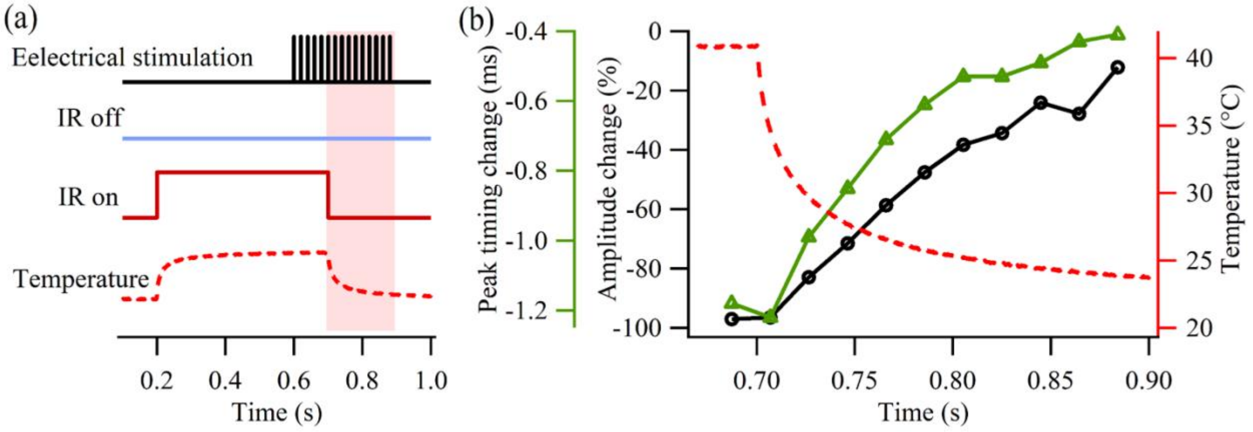

Supplementary Figure S6. Modulation of the synaptic responses after termination of the IR light illumination. (a) Illustration of the experimental protocol to evaluate the synaptic transmission during the recovery phase of the IR light-induced temperature transients (first 200 ms after the end of the IR light pulse). (b) Plot showing the normalized EPSC amplitude changes (black circles), changes in the timing of the EPSC peak (green triangles), and localized temperature transient (dashed red) caused by an IR light pulse of 13 mW during the first 200 ms of the temperature recovery phase.
